# Speeding up bioproduction of selenium nanoparticles by using *Vibrio natriegens* as microbial factory

**DOI:** 10.1038/s41598-017-16252-1

**Published:** 2017-11-22

**Authors:** Helga Fernández-Llamosas, Laura Castro, María Luisa Blázquez, Eduardo Díaz, Manuel Carmona

**Affiliations:** 1 Environmental Biology Department, Centro de Investigaciones Biológicas-CSIC, Ramiro de Maeztu 9, 28040 Madrid, Spain; 2 Department of Material Science and Metallurgical Engineering, Facultad de Ciencias Químicas, Universidad Complutense de Madrid, Av. Complutense s/n, 28040 Madrid, Spain

## Abstract

Selenium and selenium nanoparticles (SeNPs) are extensively used in biomedicine, electronics and some other industrial applications. The bioproduction of SeNPs is gaining interest as a green method to manufacture these biotechnologically relevant products. Several microorganisms have been used for the production of SeNPs either under aerobic or anaerobic conditions. *Vibrio natriegens* is a non-pathogenic fast-growing bacterium, easily cultured in different carbon sources and that has recently been engineered for easy genetic manipulation in the laboratory. Here we report that *V. natriegens* was able to perfectly grow aerobically in the presence of selenite concentrations up to 15 mM with a significant survival still observed at concentrations as high as 100 mM selenite. Electron microscopy and X-ray spectroscopy analyses demonstrate that *V. natriegens* cells growing aerobically in selenite-containing LB medium at 30 °C produced spherical electron-dense SeNPs whose size ranged from 100–400 nm. Selenite reduction just started at the beginning of the exponential growth phase and the release of SeNPs was observed after cell lysis. Remarkably, *V. natriegens* produced SeNPs faster than other described microorganisms that were proposed as model bioreactors for SeNPs production. Thus, the fast-growing *V. natriegens* bacterium becomes a suitable biocatalyst for bioremediation of selenite and for speeding-up the eco-friendly synthesis of SeNPs.

## Introduction

Selenium is a metalloid widely used in several industrial applications. In biomedicine selenium is used as supplement in the diet with healing benefits^1,2^. Due to its semiconductor and photoelectrical properties selenium is also used in electronics, photocopiers, solar cells, photography or rectifiers^3,4^. Selenium nanoparticles (SeNPs) with defined size and shape have also important biotechnological applications in electronics, cosmetics, coating and packaging^5,6^. In biomedicine, SeNPs have shown demonstrated antioxidant properties^7,8^, antitumoral and therapeutic activities against breast and lung cancer cells^6,9–11^, and antimicrobial activity against bacteria and fungi^12^. SeNPs can be synthetized by physical or chemical methods such as laser ablation, UV radiation, hydrothermal techniques, precipitation catalytic reduction, acid decomposition^13–17^. However, some of the conditions used, e.g., acidic pH or poisonous chemicals, render NPs unsafe for medical applications^16^. In general, the production of NPs using alive organism such as bacteria, fungi or plants is less expensive and safer since it uses eco-friendly non-toxic materials^6,18–22^. Biogenic SeNPs synthetized employing microorganisms have many biomedical applications^6^. In biomedicine bioproduced SeNPs has been demonstrated their antimicrobial activity against pathogenic microorganism^12,23^ being able to disrupt microbial biofilm^24^. SeNPs also show antioxidant activity since are able to scavenge reactive oxygen species^7,25^. The activity of the SeNPs is size dependent, e.g., the smallest SeNPs have the highest free radical scavenging potential^7^, being biosynthesis controlled conditions the easiest way to produce SeNPs of the desired size. Moreover, whereas the SeNPs produced by physical-chemical methods require the addition of stabilizing agents during their synthesis^26^, bioproduced SeNPs are naturally coated by organic molecules that prevent their aggregation enhancing their stability and biological, e.g. anticancer, efficiency^27,28^.

Selenium is widely distributed in nature and is present in different species depending on the environmental prevailing redox conditions^29^. The predominant oxyanions of selenium, i.e., selenite [Se(IV), SeO_3_^2−^] and selenate [Se(VI), SeO_4_^2−^], might cause severe toxicity in the environments and promote harmful effects on the cell viability^30^. Bacteria are able to greatly contribute to the interchange of the selenium forms being important elements in the selenium cycle in nature^31^. The selenium oxyanions can be reduced to elemental selenium [Se(0)], a less toxic and insoluble form. In addition, some bacteria are able to methylate selenium generating organic methylated forms, such as dimethyl selenide or dimethyl diselenide [Se^2−^]^32^. On the other hand, selenium is also an essential trace element extremely important in the physiology of the cell as part of selenocysteine, coenzyme Q, glutathione peroxidase or thioredoxin reductase^33,34^. Bacteria have developed several mechanisms of resistance to avoid the toxicity of selenium oxyanions, but the molecular basis of some of these mechanisms has not been completely elucidated yet. It has been proposed that the reduction of selenate to selenite involves the participation of reductases, e.g., the nitrate or nitrite reductases, in denitrifying bacteria such as *Ralstonia eutropha* or *Paracoccus* spp.^35^, but some bacteria, such as *Thauera selenatis* or *Desulfurispirillum indicum*, are able to use selenate as terminal electron acceptor by specific periplasmic selenate reductases^29,36^. Selenite is transformed to Se(0) by several specific or unspecific reductases, e.g., thiol-containing or gluthatione reductases, nitrite reductases, and a vast variation of small molecules and enzymatic activities, in different bacterial species^37–41^. Occasionally, the bacterial reduction of selenium oxyanions is associated with the production of SeNPs with defined size and shape^31^.

Since, as mentioned above, SeNPs have remarkable industrial and biomedical applications^42^, understanding the formation of SeNPs and developing more efficient biocatalysts and bioprocesses for the synthesis of SeNPs is of great biotechnological interest. Among the ongoing trends towards bioprocess improvement, reducing the process cycle time is one of the main target issues^43,44^. In this work we analyze the aerobic bioproduction of SeNPs by the bacterium *Vibrio natriegens*. *V. natriegens* [formerly known as *Pseudomonas natriegens* ^45^ and *Beneckea natriegens* ^46^] is a non-pathogenic fast-growing bacterium (doubling time of less than 10 minutes)^45^, easily cultured in the laboratory, able to utilize a great variety of organic substrates as source of carbon and energy^46^, and that has recently been genetically engineered and proposed for speeding up biotechnological processes^47^. Here we show that *V. natriegens* is able to reduce selenite to Se(0) producing SeNPs faster than other microorganisms proposed to be suitable bioreactors for SeNPs production. The use of *V. natriegens* as bacterial chassis may offer a significant reduction of time in an eco-friendly bioprocess to produce SeNPs. As far as we know this is the first example of the utilization of *V. natriegens* as model biocatalyst for speeding up a biotechnological process.

## Results and Discussion

### *V. natriegens* tolerates selenite

**Figure 1:**
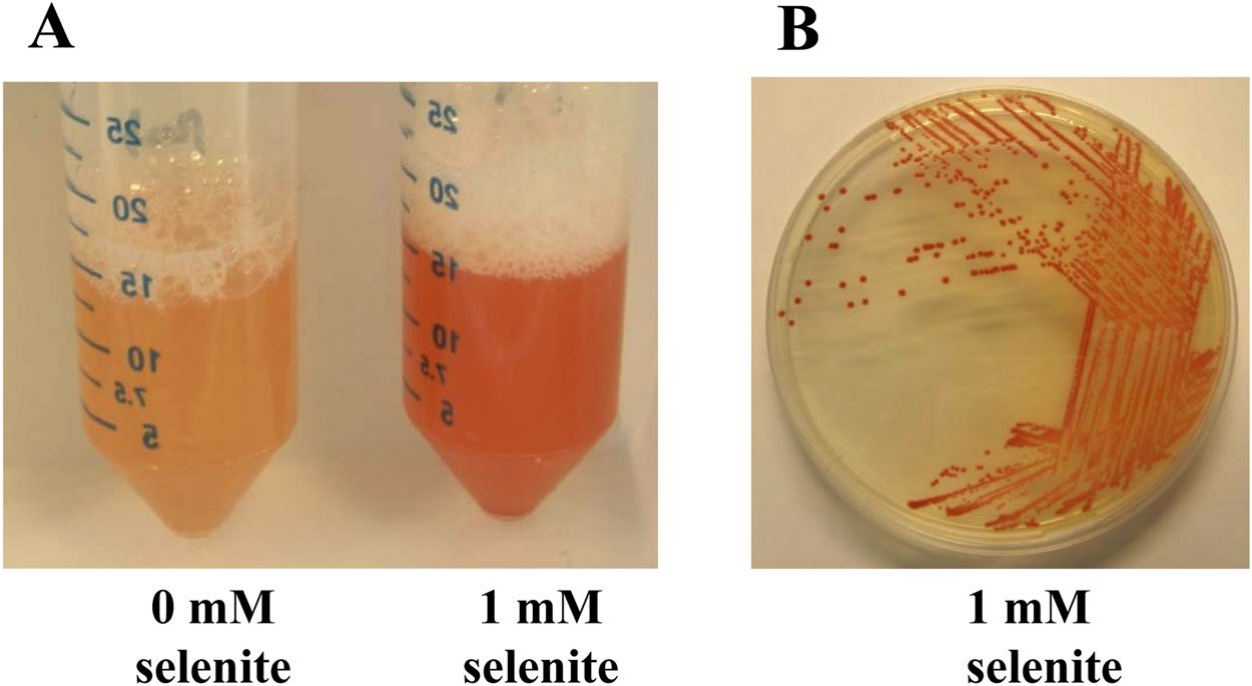
Growth of *V. natriegens* in LB broth in the presence of selenite. Liquid (**A**) and solid medium (**B**) turned to red only in the presence of 1 mM selenite. Images were obtained after culturing for 24 h.

**Figure 2:**
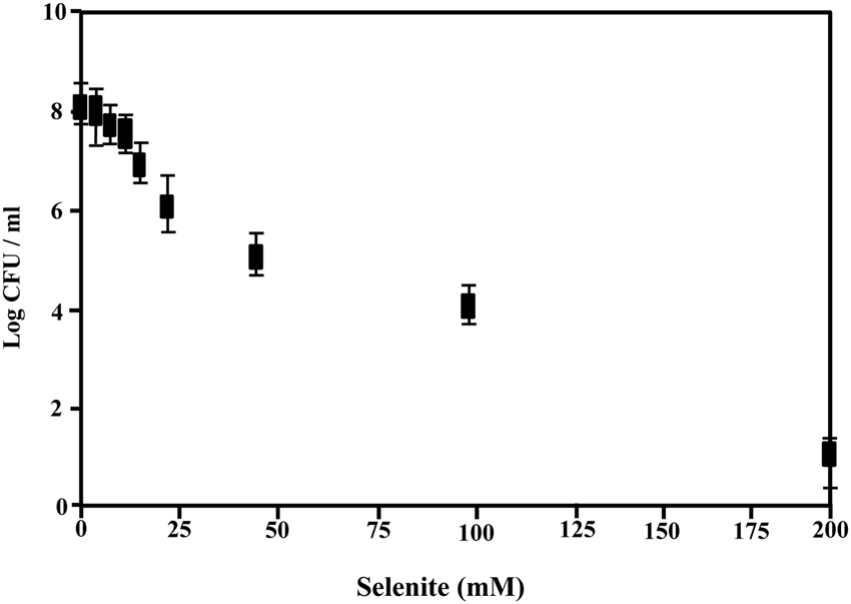
Analysis of the resistance of *V. natriegens* to selenite. Evaluation of the viability of *V. natriegens* cultures, measured as the logarithm of colony forming units (CFU) ml^−1^, grown in the presence of different selenite concentrations. Cell counting was as detailed in Methods. Error bars represent the standard deviation of at least three independent experiments.

As indicated above, *V. natriegens* has been recently proposed to be an excellent chassis for biotechnological applications due to its fast growth and, hence, rapid biosynthesis of desired compounds^47^. As a proof of concept, we analyzed whether *V. natriegens* was able to resist aerobically selenium oxyanions, such as selenate (Na_2_SeO_4_) and selenite (Na_2_SeO_3_), and to reduce them to elemental selenium Se(0), as an example of biotechnological relevant process. To this end, we grew *V. natriegens* cells in rich medium (LB) supplemented with 1 mM selenate or 1 mM selenite, respectively. After 12 hours of aerobic growth at 30 °C, the culture medium containing selenite acquired a red color (Fig. 1) that suggested the reduction of selenite to elemental selenium. No color change was observed if the selenite-containing medium was not inoculated with *V. natriegens* cells, suggesting the active participation of this bacterium in selenite reduction. Interestingly, *V. natriegens* was able to perfectly grow aerobically in the presence of selenite concentrations up to 15 mM, and a significant survival was still observed at concentrations as high as 100 mM selenite (Fig. 2). These data reveal that *V. natriegens* possesses a level of resistance to selenite much higher than that described for other bacteria (Table 1), even those widely used in environmental applications, e.g. *P. putida* KT2440^48^, and close to that reported for highly tolerant strains such *Pseudomonas moraviensis* ^49^ or *Comamonas testosteroni* S44^50^.

**Table 1:** Features of some bacteria cell cultures in the presence of selenite.

Bacteria	Selenite tolerance	Growth	SeNPs size	Selenite reduction detection*	Reference
*Bacillus mycoides* Sel TE01	25 mM	Aerobic	50–400 nm	Early-exponential growth phase (5 h)	55
*Shewanella* sp. HN-41	1 mM	Anaerobic	11–20 nm	Mid-exponential growth phase (12 h)	54
*Stenotrophomonas maltophilia* SelTE02	5 mM	Aerobic	100–300 nm	Early-exponential growth phase (80 h)	69
*Rhodopseudomonas palustri*s N	8 mM	Anaerobic	80–200 nm	Stationary growth phase (50 h)	70
*Pseudomonas moraviensis*	120 mM	Aerobic	ND	Stationary growth phase (12 h)	49
*Synechococcus leopoliensis*	5 mM	Aerobic	174–390 nm	Mid-exponential growth phase (24 h)	71
*Rhodospirillum rubrum*	1.5 mM	Anaerobic	ND	Late-exponential growth phase (70 h)	72
*Azoarcus* sp. CIB	8 mM	Anaerobic	88 ± 40 nm	Stationary growth phase (48 h)	53
*Pseudomonas putida* KT2440	10 mM	Aerobic	100–500 nm	Mid-exponential growth phase (12 h)	48
*Vibrio natriegens*	100 mM	Aerobic	100–400 nm	Early-exponential growth phase (3 h)	This work

^*^Time required to detect selenite reduction in the cell culture is indicated in brackets.

ND: not determined.

*V. natriegens* was also able to grow aerobically in the presence of selenate in the medium, however no red color was observed after 24 h of growth suggesting that selenate was not reduced to selenite and then to elemental selenium. The level of resistance to selenate was lower than that observed for selenite since concentrations of 20 mM selenate decreased the viability of the culture in more than four orders of magnitude and *V. natriegens* was not able to grow in a medium containing 50 mM selenate (data not shown).

Taken together all these results reveal for the first time that *V. natriegens* possesses an outstanding ability to tolerate, and likely reduce, selenite under aerobic conditions. To confirm and exploit this new and biotechnologically relevant property of *V. natriegens* further studies were accomplished.

### Kinetics of selenite reduction

**Figure 3:**
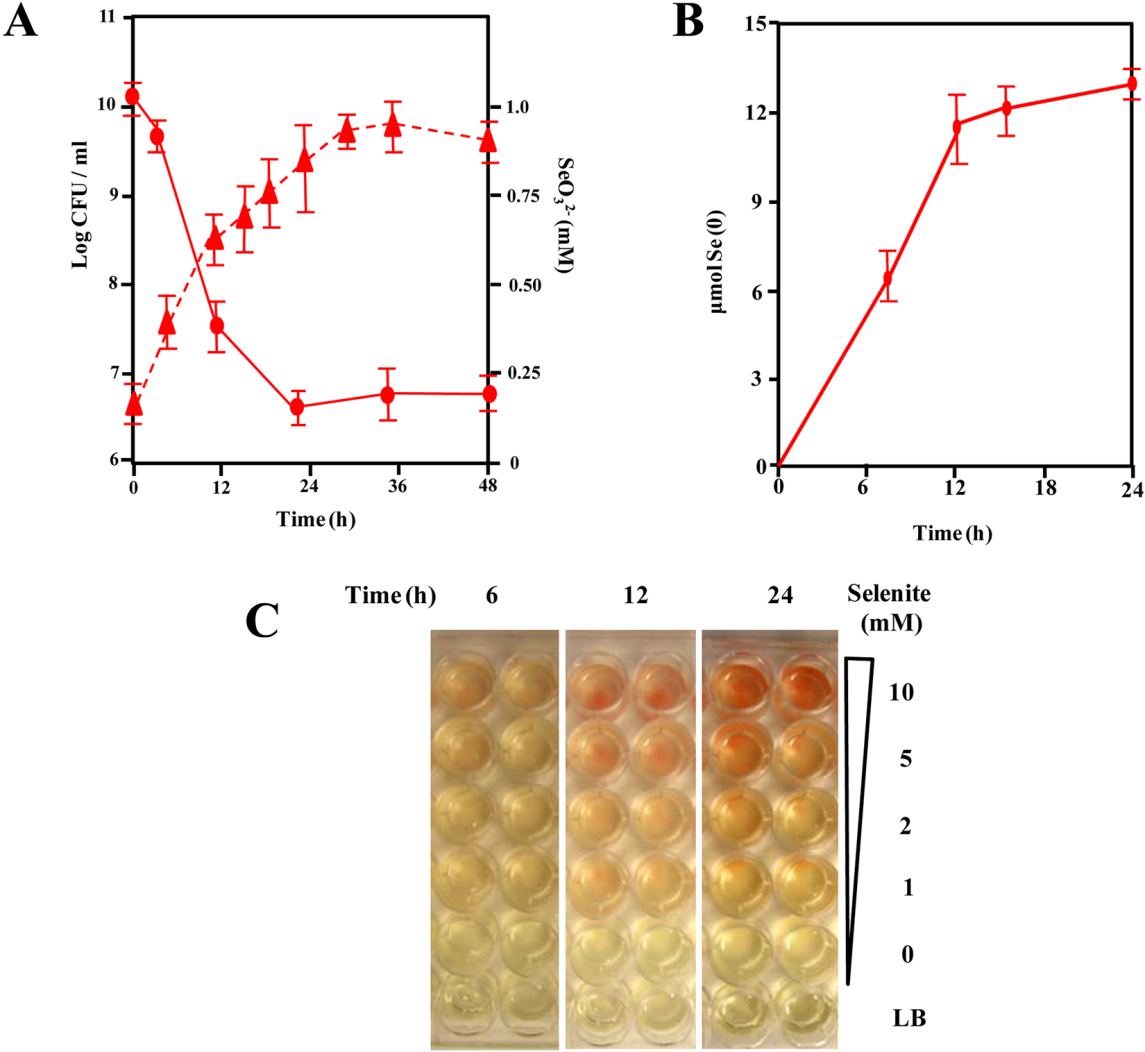
Time course of growth and selenite reduction by *V. natriegens*. (**A**) *V. natriegens* was grown in LB medium containing 1 mM selenite. Kinetics of growth, measured as CFU ml^−1^ (triangles), and selenite depletion (circles), were determined by using ICP-OES as indicated in Methods. Error bars represent standard deviation of at least three independent experiments. (**B**) Time course of Se(0) produced by *V. natriegens* (red line) cells growing in LB medium supplemented with 1 mM selenite. The amount of Se(0) was determined as indicated in Methods. Error bars represent standard deviation of at least three independent experiments. (**C**) Growth and selenite reduction (monitored by red color formation) in LB microtitre plates using selenite concentrations from 0 up to 10 mM after 6, 12 and 24 hours. LB: medium without cell inoculation.

To analyze the disappearance of selenite ions when *V. natriegens* grows in LB medium supplemented with 1 mM selenite, we used inductively coupled plasma optical emission spectrometry (ICP-OES). Remarkably, selenite disappeared for the cell culture just from the beginning of the exponential growth phase, and about 70% selenite was consumed after 12 h of growth when the number of cells increased from 5.10^6^ to 5.10^8^ CFU/ml (Fig. 3A). This interesting feature contrasts with previous reports showing that in bacterial cultures that have a significant capacity to reduce selenite to Se(0) (Table 1), e.g. *P. putida* KT2440 cultures, selenite reduction only starts at the middle-exponential growth phase and, thus, there is a significant delay of about 12 h until selenite depletion begins^48^. To confirm that selenite was reduced by *V. natriegens*, elemental selenium produced along the growth curve was measured. At 12 h of growth, *V. natriegens* produced 12 μmols of Se(0) with a rate of 1 μmol h^−1^ (Fig. 3B), thus revealing that selenite becomes reduced by the bacterial cells.

Taken advantage that *V. natriegens* possesses a high level of resistance to selenite (see above), we tested the reduction of selenite concentrations higher than 1 mM by monitoring the appearance of the red color in the culture medium. In all concentrations tested, *V. natriegens* produced the red color as fast as 12 h after inoculation (Fig. 3C), suggesting a good efficieny of selenite reduction even at high (10 mM) selenite concentrations. This result contrasts with that reported in *P. putida* KT2440, which required long incubations (48 h) to produce visible red precipitates in the presence of 10 mM selenite^48^.

Taken together all these data show that *V. natriegens* is a high selenite tolerant (Table 1) and represents the fastest biocatalyst for selenite reduction reported so far.

### Characterization of SeNPs

**Figure 4:**
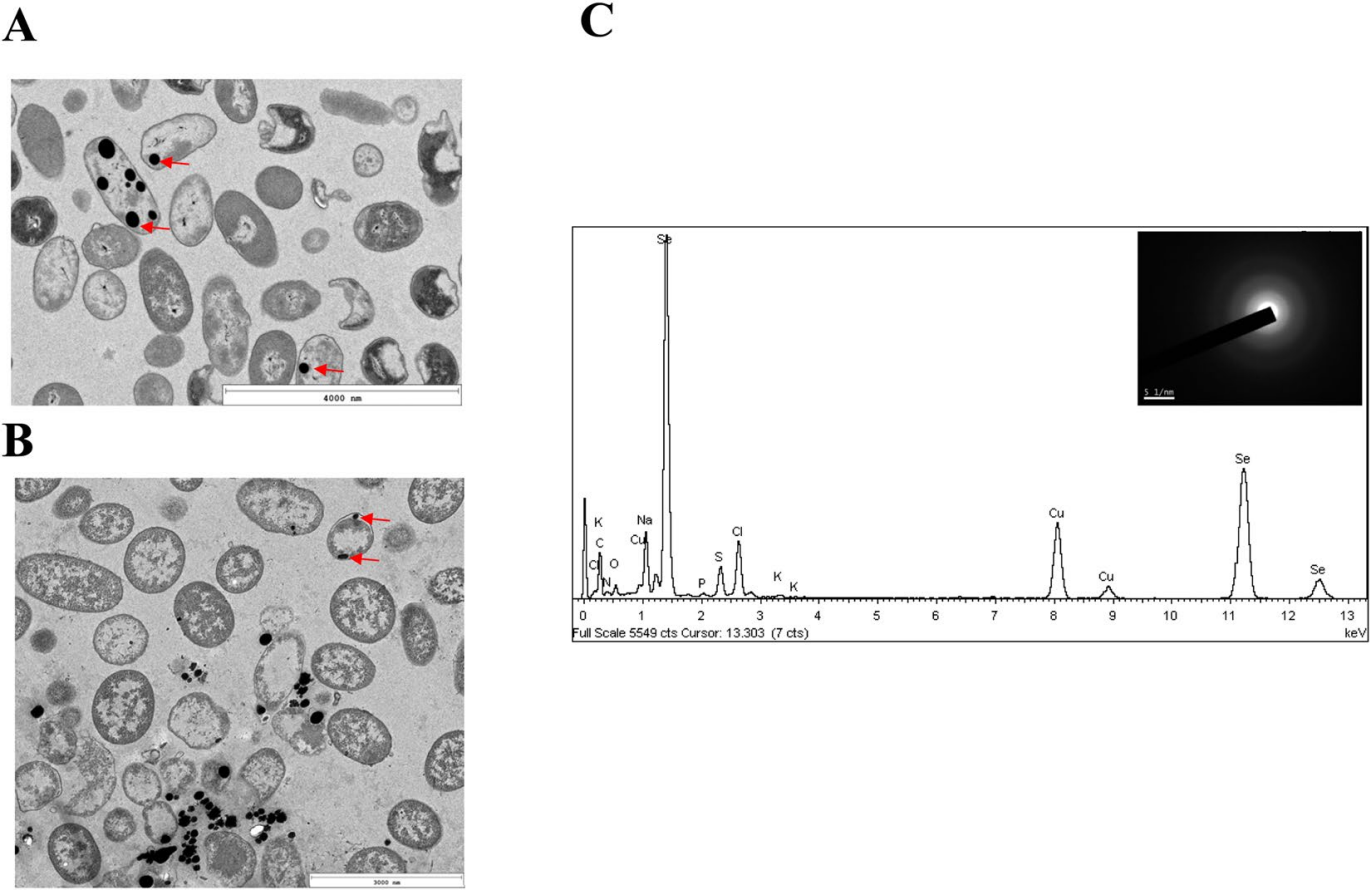
Analysis of the SeNPs production by *V. natriegens*. (**A** and **B**) TEM analysis of *V. natriegens* cells grown in the presence of 1 mM selenite showing electron-dense nanoparticles (red arrows) located intracellularly (**A**) or extracellularly (**B**) (**C**) EDX analysis of one SeNP of panel B showing its selenium composition. In the inset are shown the diffuse rings in the SAED (selected area electron diffraction) pattern of one SeNP. White line on the inset represents 5 1/nm.

Since a good number of bacteria link the reduction of selenite to elemental selenium with the production of SeNPs, we investigated whether *V. natriegens* has also the ability to convert selenite to SeNPs. To this end, we collected cells of *V. natriegens* after 24 h of growth in the presence of 1 mM selenite, and observed them using transmission electron microscopy (TEM). Electron-dense nanoparticles were obseved (Fig. 4A and B). The elemental analysis using energy-dispersive X ray spectroscopy (EDX) showed that the electron-dense particles presented the specific selenium peak (Fig. 4C). The diffuse rings in the SAED (selected area electron diffraction) pattern suggested that selenium is present in its amorphous form (Fig. 4C, inset). SeNPs were clearly observed both inside the cells (Fig. 4A) and outside of cells associated to cellular debris (Fig. 4B), suggesting that this extracellular location is most probably consequence of cell lysis. In this sense, most of the bacteria described so far that produce SeNPs release the nanoparticles after cell lysis^51^.

**Figure 5:**
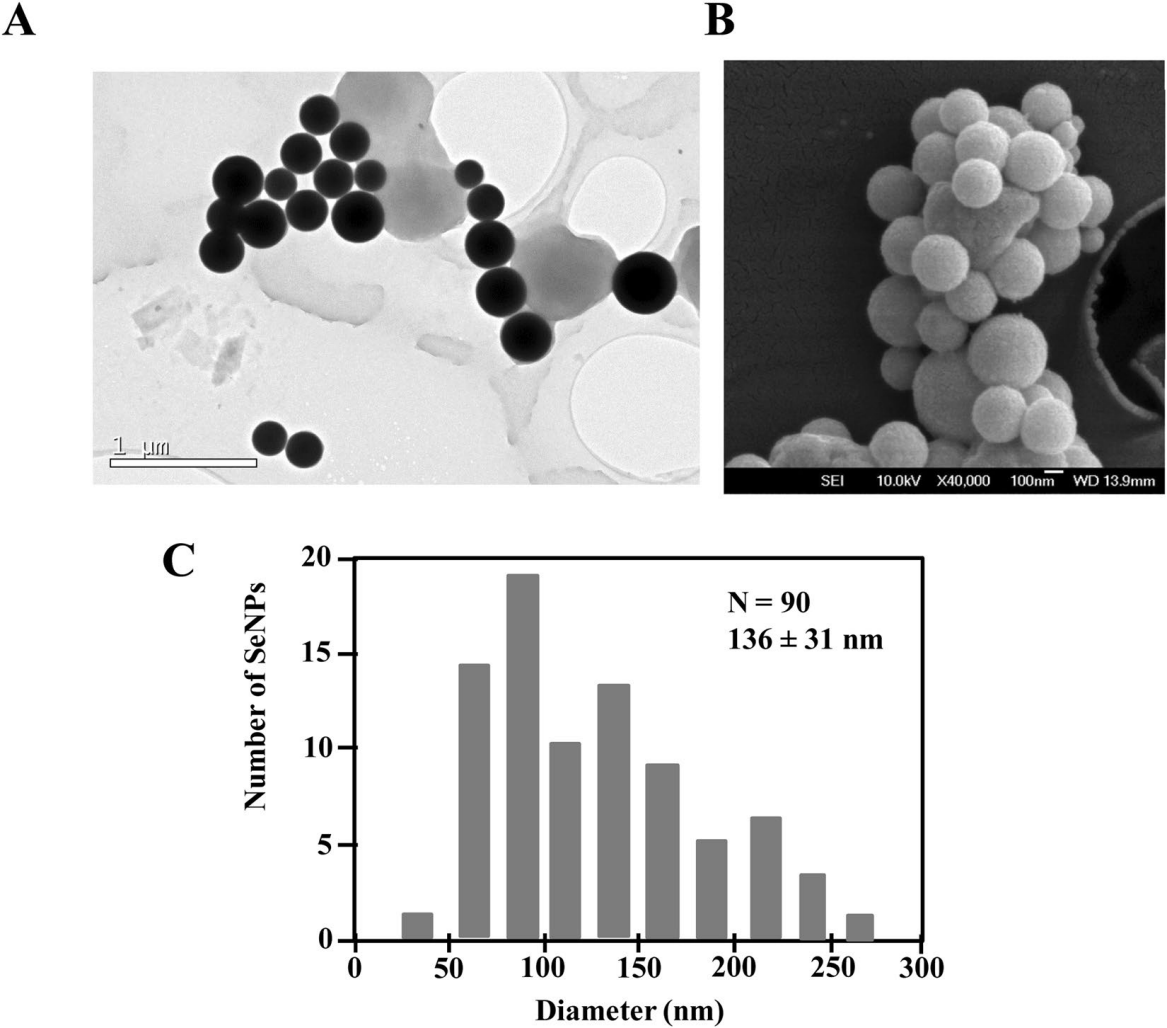
Microscopic observation and size distribution of purified SeNPs. TEM (**A**) and SEM (**B**) observation of the purified SeNPs produced by *V. natriegens* showing their spherical shape. (**C**) Size distribution of SeNPs produced by *V. natriegens*.

**Figure 6:**
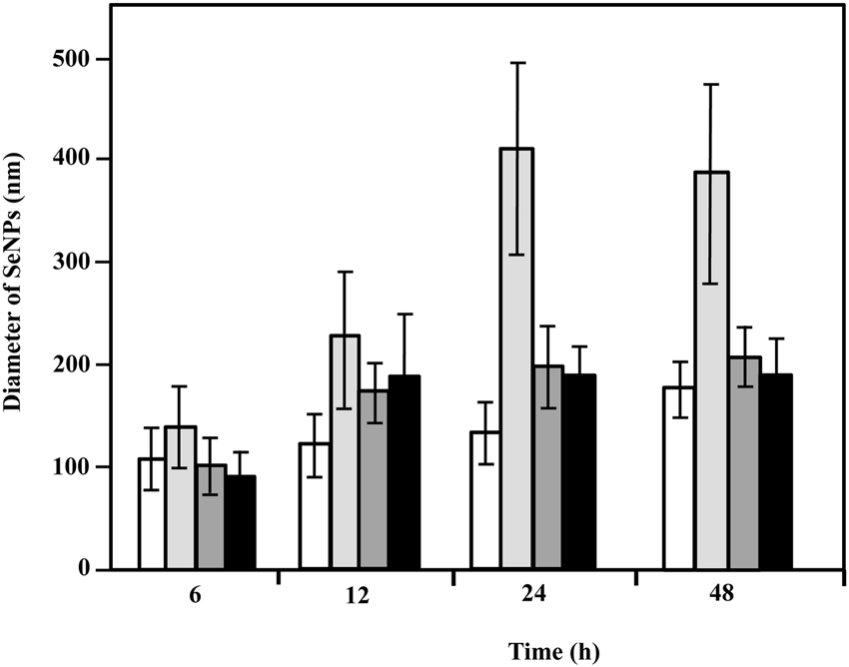
Size distribution of purified SeNPs. Estimation of the diameter of the SeNPs produced by *V. natriegens* grown at the indicated times in LB medium supplemented with 1 mM (white column), 10 mM (pale grey column), 50 mM (dark grey column) or 100 mM (black column) selenite. Error bars represent the standard deviation of at least 40 independent measurements.

The produced SeNPs were purified as described in Methods^52^ and analyzed by TEM (Fig. 5A) and scanning electron microscopy (SEM) (Fig. 5B). Purified SeNPs appeared as spherical nanoparticles with an average size of 136 ± 31 nm (Fig. 5C). Different sizes of bioproduced SeNPs have been described^53^ ranging from the 11 nm in *Shewanella* sp. HN-41^54^ to the 400 nm found in *Bacillus mycoides* Sel TE01^55^ (Table 1). Since the size of the SeNPs is an important factor that determines their chemical properties and biological activities^7^, we checked whether the size of the SeNPs produced by *V. natriegens* could be tailored by adjusting the bacterial incubation time as well as the selenite concentration used. There is a general trend showing that the SeNPs size increases when increasing the incubation time and selenite concentration (Fig. 6). Remarkably, when using 10 mM selenite and incubation times of 24–48 h the SeNPs size reaches its maximum (about 400 nm). These results reveal that the *V. natriegens-*derived bioprocess can be tuned to produce SeNPs of different defined sizes and, therefore, it constitutes a versatile platform that may be suitable for different biotechnological applications.

The mechanisms behind the formation of SeNPs in bacteria are not fully understood yet. It has been described that SeNPs accumulate within the cell (cytoplasm and/or periplasm) or in the culture medium^36^, and most bacteria accumulate SeNPs during the exponential phase and release them at the stationary phase^36^. In the well-studied *Thauera selenatis* bacterium, the SefA (selenium factor A) protein was shown to be involved in the export of the SeNPs from the cytoplasm and it helps in bio-mineralization and stabilization of the nanoparticles^36^. Besides SefA, no other protein has been so closely related with SeNPs synthesis and exportation, and most biological functions responsible of the mineralization of selenite to Se(0) that ends in a spherical chemically pure SeNP are still unknown. Nevertheless, a good number of proteins have been isolated from the SeNPs surface, and they have been suggested to be related with the mineralization process^56,57^. The knowledge of how nascent (or initially precipitated) elemental selenium coalesce to develop a true spherical SeNP is a challenge that is being approached in many laboratories^58–60^. Mathematical models and mechanisms for nucleation and growth of metal NPs, including silver and gold, have been proposed^58,61^, but these processes have not been investigated in the case of selenium^60^. Thus, *V. natriegens* might behave as a suitable model system to study the underlying biological mechanisms of SeNPs production in bacteria.

### Conclusions

The use of whole bacteria as biocatalysts is an attractive, economical and green alternative to the large scale synthesis of NPs. Several important challenges must be overcome before this green-based approach might be able to successfully compete with chemical biosynthesis^62^. An important factor is the selection of the best biocatalysts whose intrinsic properties allow to synthesize metallic NPs in a fast and efficient way^62,63^. In this sense, *P. putida* has been recently postulated as a suitable bioreactor for fast SeNPs production^48^. Here we report the use of an alternative biocatalyst, the bacterium *V. natriegens*, a non-pathogenic bacterium, easily cultured in a vast variety of carbon sources and genetically manipulable^46,47^ that shows a remarkable resistance to selenite and is able to significantly speed-up the aerobic conversion of this oxyanion for the quickest bioproduction of SeNPs so far described (Table 1). In addition, tuning the growth conditions of *V. natriegens* enable the production of SeNPs of different defined sizes and, therefore, it constitutes a versatile selenite bioconversion platform that may be suitable for different biotechnological applications.

## Methods

### Bacterial strains, culture media and growth conditions

*Vibrio natriegens* ATCC14048 was grown on LB^64^ aerobically at 30 °C with orbital shaking at 200 rpm. Solid LB medium was prepared by the addition of Bacto Agar (1.5% w/v). When appropriate, sodium selenite (Sigma-Aldrich) was added at the indicated concentration.

### Estimation of selenite tolerance

To establish the selenite tolerance of *V. natriegens*, bacterial cells were grown in LB at 30 °C with different concentration of selenite (0–200 nM). After 24 h of incubation, 1 ml of culture was used for serial dilution (form −1 to −10) and each dilution was plated on LB media. Colony forming units (CFU) were counted after 24 h of incubation at 30 °C.

### Determination of selenite concentration

**S**elenite concentration in the culture samples was determined by coupled plasma optical emission spectrometry (ICP-OES) (Perkin Elmer Optima 2100 DV)^65^.

### Determination of Se(0)

The calculation of the amount of Se(0) produced by the microbial reduction of selenite was performed following a protocol previously established^66^. Briefly, 20 ml of cell culture grown at 30 °C on LB supplemented with 1 mM selenite was collected by centrifugation at 13000 rpm during 2 min. The pellet was washed 3 times with NaCl 1 M, gently resuspended in a solution of 400 μl Na_2_S 1 M, and incubated for 1 h at room temperature. Later, the mix was centrifuged for 2 min at 13000 rpm and the absorbance at 500 nm of the supernatants was determined in the spectrophotometer. The concentration of Se(0) was estimated by interpolating in a calibration curve obtained as detailed in Biswas *et al*.^66^.

### SeNPs purification

For the purification of the SeNPs, a previously published protocol was followed^52^. This protocol is based on a separation by centrifugation of the SeNPs produced by the bacteria in an mixture composed by chloroform, ethyl alcohol and water (3:1:4).

### Characterization of SeNPs

For Transmission Electron Microscopy (TEM) analysis, the samples were prepared by placing drops of the *V. natriegens* cell cultures or the purified SeNPs onto carbon-coated copper grids and allowing the solvent to evaporate. TEM observations were performed on a JEOL model JEM-2100 instrument operated at an accelerating voltage of 200 kV. The chemical composition of the SeNPs observed was determined by energy-dispersive X-ray spectroscopy (EDX) as previously described^67^.

For field emission Scanning Electron Microscopy (SEM), the SeNPs samples were filtered through 0.2 μm pore-size filters and successively dehydrated with acetone/water mixtures of 30, 50 and 70% acetone. After critical point drying, samples were coated with graphite and gold and examined with a JEOL JSM-6330 F microscope.

The size of the SeNPs was determined by using the ImageJ software^68^.
